# Book Review: Environmental Pollutants and Their Bioremediation Approaches

**DOI:** 10.3389/fbioe.2018.00193

**Published:** 2018-12-10

**Authors:** Pankaj Chowdhary, Vishvas Hare, Abhay Raj

**Affiliations:** ^1^Department of Microbiology, Babasaheb Bhimrao Ambedkar University (A Central University), Lucknow, India; ^2^Environmental Microbiology Section, CSIR-Indian Institute of Toxicology Research, Lucknow, India

**Keywords:** constructed wetland, microbial enzyme, laccases, microalgae, nanoparticles, biochar applications

Deterioration of environmental health caused by rapid industrialization, urbanization and increasing population pressure, is a major concern for developing countries, including India. The contamination of the environment (soil/water/air) by various toxic pollutants released from several natural as well as anthropogenic activities and its adverse effects on living entities requires increased research aimed at mitigating and solving these problems effectively. In comparison to conventional remediation approaches, modern methods are more superior and effective in removing a large amount of organic and inorganic pollutants from contaminated sources (Ren and Umble, 2016). Most of environmental pollutants such as phenolics, non-phenolics, endocrine disrupting chemicals (EDCs) and heavy metals (HMs) are highly toxic in nature. Governments around the globe are strictly advocating for the mitigation of environmental pollution. For this reason, the mitigation/removal of pollutants from the environment is of utmost importance as it promotes the sustainable development of our society with a minimal environmental impact.

This book is timely and focusses on the necessity to learn about the existing environmental problems and suggests ways to control or contain their effects, by employing various treatment approaches, as well as recycling. The book consists of 15 chapters, with contributions made by many national and international professors, scientists, and researches from Egypt, Korea, and India.

In the first chapter of this book, readers gain comprehensive knowledge on several methods of bioremediation (*in-situ* and *ex-situ*) of hazardous pollutants. Chapter 2 describes in detail the persistent organic pollutants (POPs) and other toxic chemicals associated with production, use and disposal of certain organic chemicals. These chemicals are produced commercially for pest and disease control, crop production and industrial use. Chapter 3–7 of the book covers specific pollutants, from different sources which include pesticides, uranium radionuclide, dyes (acidic and basic), and lindane contamination of the environment, respectively. These chapters not only describe the adverse effects of these pollutants on the environment, but also details the remedial measures needed for the problems caused by these pollutants.

The presence of pollutants, including non-aqueous phase liquids (NAPLs), can interact with the local environment in a variety of ways, both prior to and during the oxidant treatment. These pollutants are easily mixed with water or organic solvents like oil, gasoline and petroleum products. Therefore, they affect not only terrestrial but also aquatic flora and fauna. The groundwater containing NAPL, is a complex three-phase system (i.e., water, porous media, and NAPL) and there are a variety of potential interactions that can occur, both prior to and during oxidant treatment as described in chapter 8 (McKenzie et al., 2016).

Chapters 9, 11, and 14 highlight recent advances in phytoremediation as well as the role of bacterial ecology of the plant rhizosphere in the bioremediation of industrial effluents from the distilleries, pulp & paper and textile industries, etc. (Chowdhary et al., 2017). Several naturally growing plant species have the inherent capacity to uptake and accumulate HMs in different plant parts (root, shoot, leaf and fruits), whereas some other species also participate in the biodegradation and biotransformation/conversion of more toxic pollutants, to less toxic forms and therefore assist in sustainable development. The phytoremediation/rhizoremediation of toxic HMs is clearly described in terms of plant mechanisms, to remove or mitigate pollutants from the contaminated sites. This process involves the use of microorganisms, green plants (*Typha latifolia, Phragmites australis, Saggittaria latifolia, Phragmites communis*, and *Juncus effuses* etc.) or their enzymes, to degrade/detoxify the environmental pollutants such as toxic metals, pesticides, azo dyes, petroleum hydrocarbons, plastics, phenols, chlorophenols, biomedical wastes etc. from the polluted soils and water resources (Chandra and Chowdhary, 2015). Microbial cellulases have shown their potential application in various industries like pulp and paper, laundry, textile, bioethanol production, brewing, food and feed industry. The waste generated from agro-industries, agricultural waste, and forests contain huge amount of unused cellulose causing an adverse impact on environment. Their complex nature and wide spread industrial applications are detailed in chapter 10.

Chapters 12, 13, and 15 describe various types of waste such as solid waste, tannery waste and biomedical waste, respectively. When we think about their management, the first thing to come to mind is solid waste (garbage) and the amount of waste that is dumped in landfills or is incinerated. A variety of approaches are required to manage and develop a solid waste management system. Tannery industries are key contributors in the economy of many developing countries, but unfortunately, they are also sources of major pollutants globally. They release large volumes of hazardous wastewater, with unpleasant odors and a mixture of organic (benzyl butyl phthalate, di butyl phthalate, anthracene, and nonyl phenol etc.) and inorganic pollutants (chromium, lead, copper and nitrates etc.). This waste water discharge reduces sunlight penetration in aquatic resources, decreasing both photosynthetic activity and dissolving oxygen concentrations, greatly affecting aquatic life. Additionally, on land, waste water discharge causes decreased soil alkalinity and the inhibition of seed germination.

Biomedical waste (BMW) includes all the waste generated from hospitals, medical, healthcare centers and research facilities in the diagnosis, treatment, immunization and allied research field. BMW, if not treated or managed adequately, may lead to the spread of diseases such as HIV, Hepatitis B & C and other microbial diseases which may cause a hazardous impact on human health. Additionally, BMW disposal is a major concern and its safe disposal is a priority. The degradation and detoxification potential of microbes and their interactions with environmental pollutants have been studied in last few years, but detailed knowledge on the various types of environmental pollutants, their toxicological effect on environment, humans, animals and plants as well as various bioremediation approaches for their degradation/detoxification, are not collectively available. The present book covers all of these aspects lucidly and succinctly.

However, barring one lacuna, which could have been covered by including a chapter on the role of nanoparticles in pollutant mitigation, environmental education and waste management, as well as the appropriate technology needed and used for waste management, this book provides a comprehensive perspective on bioremediation & environmental sustainability. This book is highly recommended to a diverse community of professionals, scientists, students, researchers etc. who have an interest in bioremediation technologies and sustainable development.

## Author Contributions

PC helped with the writing and compilation of the manuscript, whereas VH and AR assisted in the final editing of the book review.

### Conflict of Interest Statement

The authors declare that the research was conducted in the absence of any commercial or financial relationships that could be construed as a potential conflict of interest.
